# DW2008S and its major constituents from *Justicia procumbens* exert anti‐asthmatic effect via multitargeting activity

**DOI:** 10.1111/jcmm.13550

**Published:** 2018-03-07

**Authors:** Jihyun Youm, Hyunyong Lee, Youngwoo Choi, Joobyoung Yoon

**Affiliations:** ^1^ Research Institute Dong‐Wha Pharmaceutical Company Yong‐In City Korea; ^2^ College of Pharmacy Chungnam National University Daejeon City Korea; ^3^ Department of Allergy & Clinical Immunology Ajou University School of Medicine Suwon City Korea

**Keywords:** adenosine receptor A_3_, DW2008S, *Justicia procumbens*, justicidin, phosphodiesterase 4, tyrosine‐based inhibition motif domains

## Abstract

Our previous study revealed that the ethanolic extract of *Justicia procumbens* ameliorates ovalbumin‐induced airway inflammation and airway hyper‐responsiveness in a mouse model of asthma. However, the mechanism of action of the extract remains unknown. In this study, we prepared DW2008S, an optimized and standardized powder extracted from *J. procumbens* using anhydrous ethanol, and investigated its anti‐asthmatic effect and mechanism of action. Our results showed that DW2008S contains two major ingredients, justicidin A (JA) and justicidin B (JB), which selectively inhibit T helper 2 (Th2) cell responses in concanavalin A‐activated spleen cells and polarized Th2 cells. Blockade of T cell immunoreceptor with immunoglobulin and immunoreceptor tyrosine‐based inhibition motif domains (TIGIT) using a neutralizing antibody also selectively inhibited Th2 cell responses. Furthermore, DW2008S regulated TIGIT expression in the mice and cultured cells. Additionally, DW2008S and JA antagonized human adenosine receptor A_3_ (A_3_ AR), which mediates mast cell‐dependent inflammation and bronchoconstriction. DW2008S and JB inhibited human phosphodiesterase 4 (PDE4), which is known to cause bronchoconstriction; however, the required concentrations were higher than those needed to affect TIGIT . These findings suggest that DW2008S can potentially ameliorate Th2‐driven airway inflammation and bronchoconstriction through negative regulation of TIGIT and blockade of A_3_ AR and PDE4 activities.

## 1 INTRODUCTION

According to a recent report from the Global Initiative for Asthma, asthma is a heterogeneous chronic respiratory disease affecting 1%‐18% of the global population; it is characterized by chronic airway inflammation with variable expiratory airflow limitation.^1^ Asthma, especially early‐onset asthma, is mainly caused by allergen‐specific T helper 2 (Th2)‐type responses. There is evidence that Th2 immunity plays a crucial role in inflammation and airway hyper‐responsiveness (AHR) in mouse models of allergic asthma.^2^ Th2‐type cytokines such as interleukin (IL)‐4, IL‐5 and IL‐13 stimulate the production of allergen‐specific immunoglobulin (Ig) E in B cells and infiltration of eosinophils into the lungs. This results in the release of various substances, such as histamine, leukotriene and prostaglandin *D*_2_, through degranulation of mast cells, causing mucus production and bronchoconstriction.^3^, ^4^, ^5^, ^6^ Thus, many studies have developed antibodies for the treatment or prevention of allergic asthma by targeting Th2 immune pathophysiological factors.^7^, ^8^, ^9^, ^10^ However, the clinical efficacies of these drugs are limited to alleviation of the condition. Moreover, reduction in the steroid dose is usually required because of poor improvement in lung function in patients with severe asthma.^11^, ^12^ The limited efficacies of these drugs may be due to their partial inhibition of Th2 responses. Thus, agents that target key molecules that negatively regulate Th2 cell differentiation or total Th2 responses must be developed. T cell immunoreceptor with Ig and immunoreceptor tyrosine‐based inhibition motif domains (TIGIT) is expressed on natural killer cells, memory T cells, activated T cells and regulatory T cells (Tregs). Furthermore, it is considered a novel immune checkpoint molecule.^13^ TIGIT negatively regulates T cell responses through the effect of cluster of differentiation (CD) 112 or CD155 on dendritic cells or through intrinsic inhibitory effects such as inhibition of T cell proliferation or suppression of cytokine production in CD4^+^ T cells.^14^ Recently, TIGIT^+^ Tregs have been shown to selectively suppress Th1 and Th17 responses but not Th2 responses.^15^ Moreover, TIGIT improves Th2 responses and allergic disease through the activity of CD155, whereas blockade of TIGIT with a neutralizing anti‐TIGIT antibody exerts a therapeutic effect on allergic airway inflammation in a murine model of asthma.^16^ However, there is still no chemical agent or herbal medicine that can selectively regulate Th2‐driven allergic inflammation via TIGIT regulation.

Some molecules that regulate bronchoconstriction, such as adenosine receptors (ARs) and phosphodiesterases (PDEs), are useful for alleviating asthma and Th2‐driven airway inflammation. Adenosine levels increase in the lungs of patients with allergic asthma, which contributes to bronchoconstriction and mast cell degranulation.^17^ A_2A_ AR agonists, as well as A_1_ AR, A_2B_ AR, and A_3_ AR antagonists, show anti‐asthmatic effects by reducing inflammation, bronchoconstriction and mucus secretion in animal models.^18^ A_2B_ AR and A_3_ AR have been evaluated as important targets in the development of drugs for asthma treatment because of safety issues with other ARs.^19^, ^20^

PDE3 and PDE4 are involved in respiratory diseases. It is reported that PDE3, PDE4 and mixed PDE3/4 inhibitors significantly suppress allergen‐induced contractions in passively sensitized airways in humans.^21^ Thus, roflumilast, a selective PDE4 inhibitor that reduces exacerbation frequency and significantly improves pulmonary function, is used to treat patients with severe chronic obstructive pulmonary disease (COPD).^22^

In a previous study, we showed that an anhydrous ethanolic extract of *Justicia procumbens* (DW2008) protected against allergic asthma by reducing the expression of Th2‐type cytokines and alleviating methacholine‐induced AHR in a mouse model of ovalbumin (OVA)‐induced asthma.^23^ However, the mechanisms underlying these effects remain unknown. In this study, we prepared DW2008S powder, which has better solubility and homogeneity characteristics than DW2008 has, and investigated its anti‐asthmatic effect and mechanisms of action.

## 2 MATERIALS AND METHODS

### 2.1 Materials

Chemically synthesized justicidin A (JA; batch #JA16001; purity, >95%) and justicidin B (JB; batch #JB16001; purity, >95%) were purchased from U‐Chem (Anyang, Korea). OVA, montelukast and dexamethasone were purchased from Sigma‐Aldrich (St. Louis, MO, USA). Anti‐TIGIT neutralizing antibody and a corresponding sheep IgG isotype control antibody were purchased from R&D Systems (Minneapolis, MN, USA).

### 2.2 Preparation of DW2008S powder

DW2008S was prepared from an anhydrous ethanolic extract of *J. procumbens* and colloidal silicon dioxide (1:1). *J. procumbens* was collected from Jecheon City and authenticated at the National Institute of Biological Resources, Korea (voucher specimen number: NIBRVP0000530740‐742). Dried *J. procumbens* was subjected to extraction with anhydrous ethanol (10× solvent volume), after which the filtrate was concentrated at 60°C under vacuum. The sample was dried in a vacuum oven for 12 hours in the presence of colloidal silicon dioxide. Approximately 80 kg of DW2008S (Batch No. p16001) was produced for preclinical toxicity testing and this study.

### 2.3 High‐performance liquid chromatography (HPLC) analysis

HPLC analysis of DW2008S was performed as previously described 23 using an Agilent 1200 series HPLC system (Agilent Technologies, Santa Clara, CA, USA). Data were collected and processed using OpenLAB chromatography software. Ultraviolet (UV) detection was performed at 256 nm, and the injection volume was 10 μL. Peaks detected on the chromatogram for DW2008S were identified by comparing their retention times and UV spectra to those of pure compounds.

### 2.4 Cell culture and Th cell differentiation

Spleen cells were collected from BALB/c mice as previously described.^23^ Viable spleen cells were plated at a density of 5 × 10^6^ cells/mL and cotreated with 5 μg/mL concanavalin A and test drugs for 48 hours. The levels of cytokines in the culture supernatants were measured using enzyme‐linked immunosorbent assay (ELISA) kits (Koma Biotech, Seoul, Korea).

For in vitro Th2 and Treg polarization, CD4^+^ T cells were isolated from spleen cells using a CD4^+^ T cell isolation kit (Miltenyi Biotec, Auburn, CA, USA). Next, the cells were plated at a density of 2.5 × 10^5^ cells in 0.5 mL on anti‐CD3/CD28 antibody pre‐coated plates with 20 ng/mL recombinant mouse IL‐2 (BioLegend, San Diego, CA, USA). For Th2 polarization, 100 ng/mL recombinant mouse IL‐4 (BioLegend), 10 μg/mL anti‐interferon‐γ (IFN‐γ) and 10 μg/mL anti‐IL‐12 were added to the cells, followed by incubation of the plates for 72 hours. Next, the cells were transferred into new wells without anti‐CD3/CD28 antibody, and the medium was replaced with one containing neutralizing antibodies (no cytokines). After 48 hours of incubation, fresh anti‐CD3/CD28 antibody was added to the cells and incubation was continued for 48 hours. For Treg polarization, 5 ng/mL recombinant mouse transforming growth factor‐β1 (R&D Systems) was added to the culture for 96 hours.

### 2.5 Mouse model of asthma

All animals were treated according to the Guide for the Care and Use of Laboratory Animals developed by the Institute of Laboratory Animal Resources, Commission on Life Sciences, National Research Council. The study was approved by the Institutional Animal Care and Use Committees of the Research Institute of Dong‐Wha Pharmaceutical Company and Ajou University School of Medicine.

**Figure 1:**
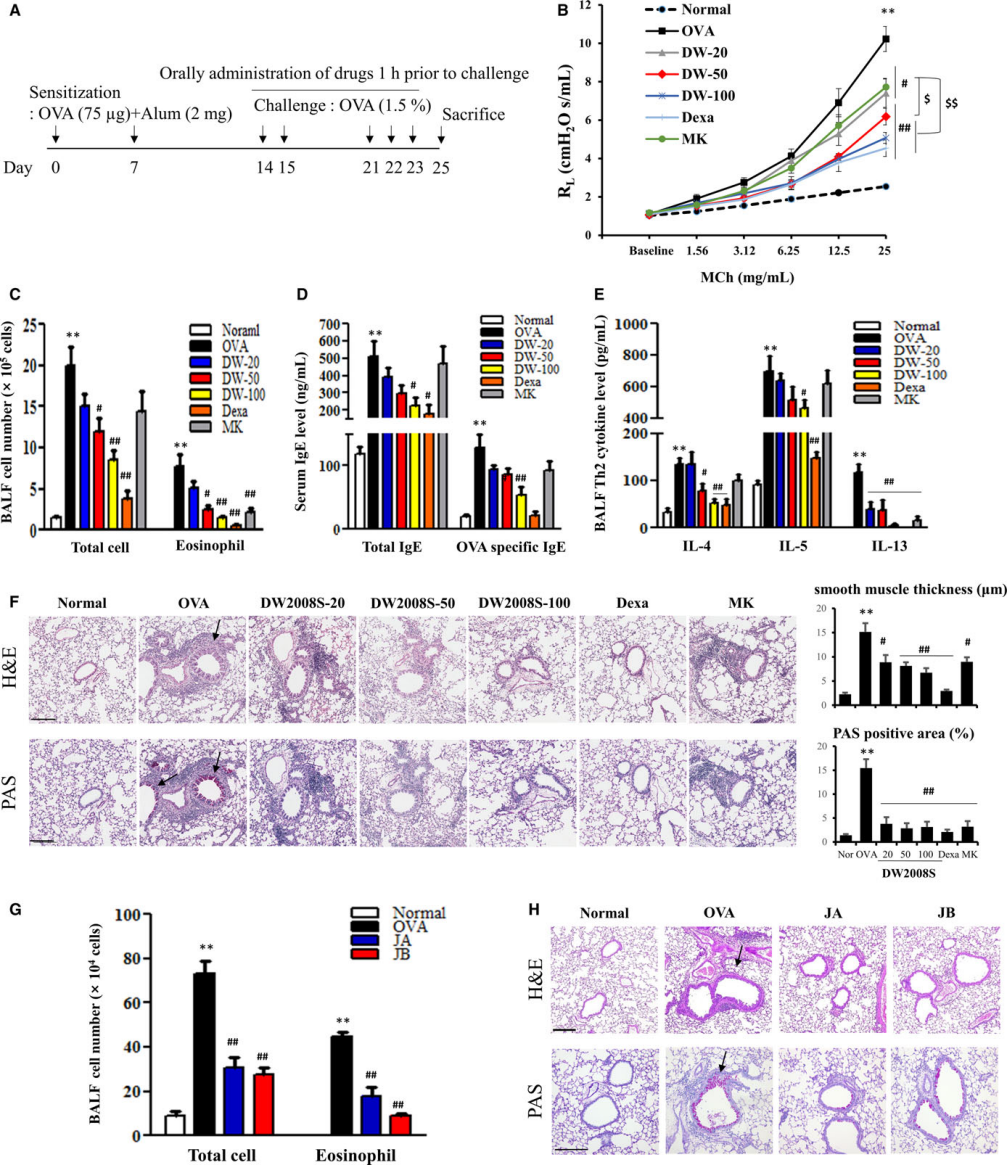
Effects of DW2008S, JA and JB on allergic airway inflammation in a mouse model of asthma. (A) Asthma mouse model scheme. (B) AHR. (C, G) Differential cell counting. (D) Serum total IgE and OVA‐specific IgE levels. (E) Protein levels of IL‐4, IL‐5 and IL‐13 in BALF. (F, H) Histology: H&E staining (upper panel) and PAS staining (lower panel). Scale bar = 200 μm. The arrows point to smooth muscles or PAS‐positive areas. All data are expressed as mean ± SE (n* *=* *5). ** indicates *p* < .01 when compared to the Normal group, # indicates *P* < .05 when compared to the OVA group, ## indicates *P* < .01 when compared to the OVA group, $ indicates *P* < .05 when compared to the MK group, and $$ indicates *P* < .01 when compared to the montelukast (MK) group. Abbreviations: Alum, aluminium hydroxide; DW, DW2008S at the indicated dose (mg/kg); Nor, Normal; MCh, Methacholine; Dexa, Dexamethasone

Six‐week‐old female BALB/c mice were acclimated to the experimental conditions for 7 days and randomly assigned to treatment groups. The mice were sensitized with an intraperitoneal injection of saline containing OVA/aluminium hydroxide and then challenged with aerosol OVA using nebulizers (Figure 1A). On the indicated days, the mice were administered DW2008S, montelukast and dexamethasone for 1 hour prior to a challenge with aerosol OVA. Serum was collected and stored at −70°C until analysis. Serum levels of total IgE and OVA‐specific IgE were determined using ELISA kits (Koma Biotech). Bronchoalveolar lavage fluid (BALF) was collected by lavaging the trachea twice with a total of 1 mL of 1× Hank's balanced salt solution. Cells were pelleted by centrifuging BALF at 400 *g* for 5 minutes. The cells were resuspended in 0.5 mL of phosphate‐buffered saline, after which total cell count was determined using a haemocytometer.

For differential cell counting, 0.1 mL of the cell suspension was cytospun onto a microscopic slide at 800 *g* for 3 minutes. The cells were then stained using Diff‐Quik^®^ staining solution kit (Sysmex, Kobe, Japan). Images were obtained from random areas, and differential cell counts were performed for >200 total cells in 1‐6 images. The levels of IL‐4, IL‐5 and IL‐13 in BALF were determined using ELISA kits (R&D Systems).

For histology, lung tissues were harvested, fixed in 10% formalin, embedded in paraffin, sectioned, mounted onto microscopic slides and then stained with haematoxylin (Dako, Carpinteria, CA, USA) and eosin (Sigma‐Aldrich) (H&E) or periodic acid‐Schiff (PAS) stain. Images of lung tissues were captured at 200× magnification. Smooth muscle thickness (H&E) and mucus‐positive areas (PAS) were analysed using ImageJ software (National Institutes of Health, Bethesda, MD, USA).

Airway resistance to inhaled methacholine was measured using a flexiVent^®^ system (SCIREQ, Montreal, QC, Canada). At 48 hours after the last challenge, mice were anaesthetized and their tracheas were exposed and intubated with cannulas. Methacholine chloride (Sigma‐Aldrich) was dissolved in normal saline (1.56‐25 mg/mL) and administered to the mice in increasing doses (0, 1.56, 3.12, 6.25, 12.5 and 25 mg/mL). Airway resistance was recorded after each dose was administered.

Mediastinal lymph nodes (MLNs) were isolated from normal and asthmatic mice as previously described^24^ and crushed for isolation of MLN cells.

### 2.6 Flow cytometry

Spleen cells, polarized CD4^+^ T cells, BALF cells and MLN cells were stained with anti‐CD4 (clone GK1.5) and anti‐TIGIT (clone GIGD7) antibodies. For intracellular staining, cells were fixed and permeabilized using Foxp3 Transcription Factor Staining Buffer kit (eBioscience, San Diego, CA, USA). Next, the cells were stained with anti‐GATA‐binding protein 3 (GATA3, clone 16E10A23), anti‐forkhead box P3 (FOXP3, clone FJK‐16s) or anti‐IL‐4 (clone 11B11, eBioscience) antibodies. All stained samples were analysed using FACSCalibur^™^ flow cytometer (BD Biosciences, Franklin Lakes, NJ, USA).

### 2.7 Quantitative reverse transcription‐polymerase chain reaction (RT‐qPCR)

Total RNA was extracted from BALB/c mouse spleen cells using TRIzol^®^ reagent (Life Technologies, Carlsbad, CA, USA) and reverse transcribed using PrimeScript^™^ RT Master Mix (TaKaRa, Shiga, Japan). cDNA levels of target genes were quantified using LightCycler^®^ 480 II Real‐Time PCR System (Roche Diagnostics, Rotkreuz, Switzerland) and SYBR^®^ Premix Ex Taq^™^ II kit (TaKaRa). Primers were designed using Primer‐BLAST program (National Center for Biotechnology Information, Bethesda, MD, USA). The primers were synthesized by Bioneer (Daejeon, Korea). The primer sequences were as follows: TIGIT: forward, 5′‐CCACAGCAGGCACGATAGAT‐3′, and reverse, 5′‐TCGACTTGGGTCACTTCAGC‐3′; β‐actin: forward, 5′‐CTAAGGCCAACCGTGAAAAG‐3′, and reverse, 5′‐ACCAGAGGCATACAGGGACA‐3′. Transcript levels of target genes were normalized to those of β‐actin and expressed as fold changes relative to the indicated controls.

### 2.8 Target screening

All target screening tests were performed by Eurofins Panlabs, Inc. (St. Charles, MO, USA; Taipei, Taiwan). The activities of G‐protein‐coupled receptors related to inflammation and asthma were determined by the GPCRProfiler^®^ Service (Cat. No. HTS600GPCR) using a fluorescence imaging plate reader (FLIPR) calcium flux assay kit. The activities of lipoxygenases (Cat. Nos. 199016, 137010, and 199017) and xanthine oxidase (Cat. No. 198000) were measured by spectrofluorimetric assays, whereas the activities of human recombinant protein tyrosine kinases (LYNA, Cat. No. 176050; LYNB, Cat. No. 176070) were measured by quantifying [^32^P]‐poly(Glu:Tyr) in insect cells. Furthermore, the activities of PDE isoforms were measured using radiolabeled substrates (Cat. Nos. 148000, 152000, and 154000), whereas the activities of A_3_ AR were additionally characterized by a radioligand‐binding assay using [^125^I]‐4‐aminobenzyl‐5′‐*N*‐methylcarboxamidoadenosine (AB‐MECA; Cat. No. 200720).

### 2.9 Statistical analysis

All data were statistically analysed using SPSS for Windows (release 10.0.7; SPSS Inc., Chicago, IL, USA). The results are expressed as mean ± standard error of the mean (SE). Variance homogeneity was evaluated using Levene's test. Data were analysed by one‐way analysis of variance. Dunnett's post hoc test was used with a homogenous variance, whereas the Mann‐Whitney *U* test was used in cases of unequal variances. *P* values <.05 were considered statistically significant.

## 3 RESULTS

### 3.1 DW2008S ameliorates Th2‐driven inflammation

The composition of DW2008S was determined by HPLC. Two major constituents, JA and JB, were identified with retention times of 50.9 and 47.3 minutes, respectively (Figure S1). To determine the efficacy of DW2008S against allergic airway inflammation, a mouse model of allergic asthma was established (Figure 1A).

In the OVA asthma group treated with DW2008S, a dose‐dependent reduction in methacholine‐induced AHR was observed. At a dose of 20 mg/kg, DW2008S reduced AHR to a level equivalent to that obtained with a saturation dose (10 mg/kg) of montelukast, a positive control that antagonizes the leukotriene receptor. Furthermore, DW2008S at doses of 50 and 100 mg/kg significantly inhibited AHR. It was observed that the extent of inhibition was higher than that caused by montelukast and equivalent to that caused by dexamethasone, a representative glucocorticosteroid (Figure 1B).

The DW2008S‐treated group showed lower infiltration of inflammatory cells and eosinophils into the lungs than the OVA asthma group did (Figure 1C). Additionally, DW2008S reduced serum total IgE and OVA‐specific IgE levels in a dose‐dependent manner (Figure 1D). Furthermore, the levels of Th2 cytokines (IL‐4, IL‐5 and IL‐13) were all significantly reduced in BALF by DW2008S (Figure 1E). Figure 1F shows that DW2008S reduced mucus‐positive areas and the thickness of airway smooth muscles in the lung tissues of the animals.

The results showed that JA and JB individually reduced the infiltration of total inflammatory cells and eosinophils into the lungs (*P* < .05 in each case, Figure 1G). Additionally, they inhibited increase in airway epithelium thickness and mucus production in the lung tissues (Figure 1H). The results indicate that at doses >50 mg/kg, DW2008S shows an anti‐asthmatic effect that is superior to that of montelukast in a mouse model of asthma. In addition, our findings confirm that JA and JB are active constituents in DW2008S.

**Figure 2:**
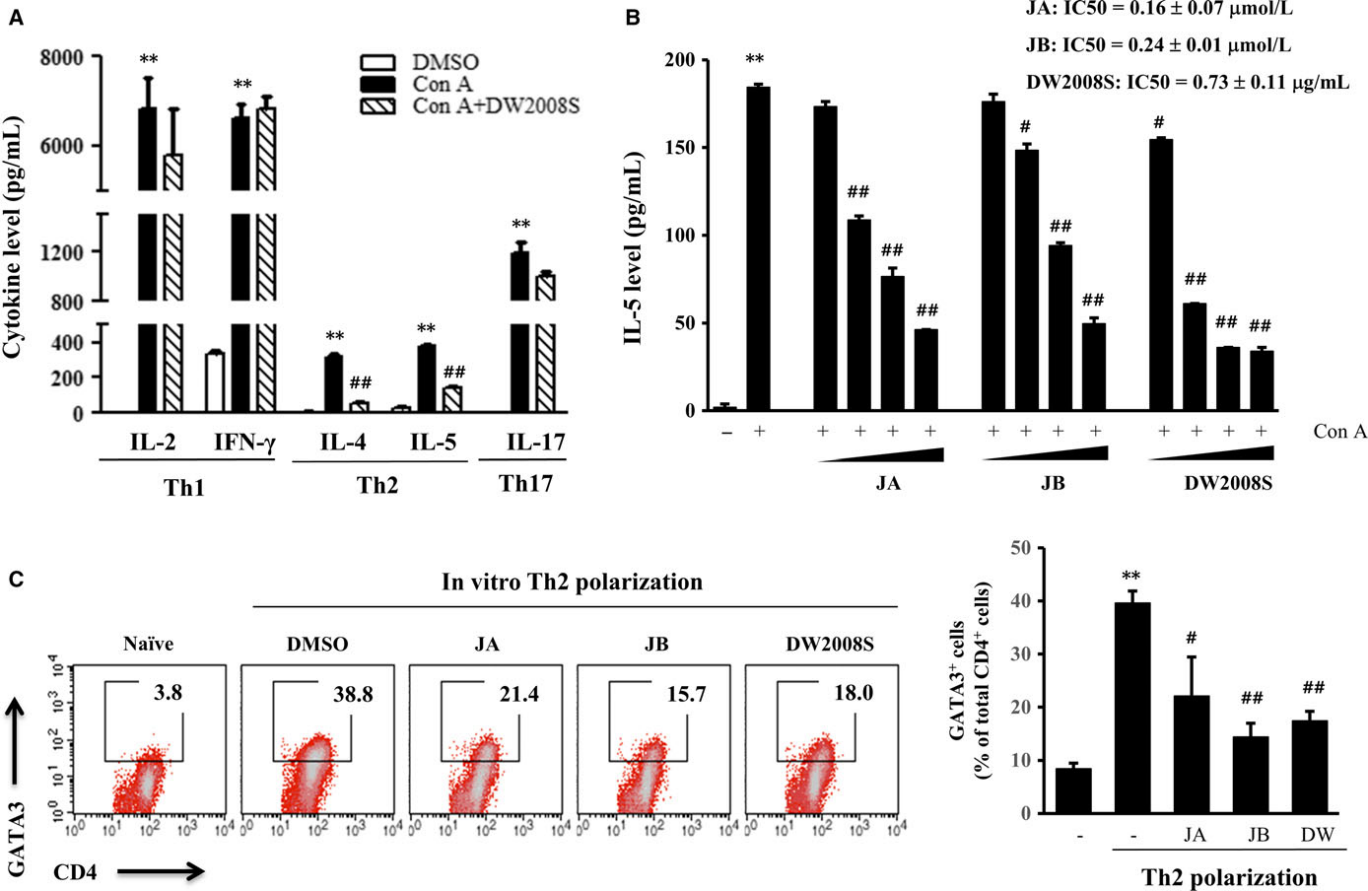
Selective inhibitory effects of DW2008S, JA and JB on Th2 responses. (A) Spleen cells were cotreated with 5 μg/mL concanavalin A (Con A) and 5 μg/mL DW2008S for 48 h. Th1/Th2/Th17 cytokine levels in the culture medium were determined using ELISA kits. (B) Spleen cells were cotreated with 5 μg/mL Con A and JA (0.05, 0.1, 0.25, or 0.5 μmol/L), JB (0.05, 0.1, 0.25, or 0.5 μmol/L), or DW2008S (0.5, 1, 2.5, or 5 μg/mL) for 48 hours. IL‐5 level was determined using an IL‐5 ELISA kit. (C) Isolated CD4^+^ T cells were treated with 0.5 μmol/L JA, 0.5 μmol/L JB or 2 μg/mL DW2008S under in vitro Th2 polarization conditions. Representative fluorescence‐activated cell sorting plots for GATA3^+^ Th2 cells and graphs plotted from the results of three independent experiments are shown. Data are expressed as mean ± SE. ** indicates *P* < .01 when compared to the vehicle, # indicates *P* < .05 when compared to Con A or the polarized Th2 sample, and ## indicates *P* < .01 when compared to Con A or the polarized Th2 sample. DMSO, dimethyl sulfoxide

To elucidate the mechanism underlying the inhibitory effects of DW2008S and its active constituents in the mouse model of allergic asthma, we evaluated the levels of Th1/Th2/Th17 cytokines in spleen cells. The results showed that concanavalin A, which is a T cell activator, increased all Th1/Th2/Th17 cytokine levels in the spleen cells. In addition, DW2008S selectively reduced the levels of Th2‐type cytokines (IL‐4 and IL‐5); however, it had little effect on Th1‐type cytokine (IFN‐γ) levels in concanavalin A‐activated spleen cells. IL‐17 is a signature Th17 cytokine. The results showed that IL‐17 level was slightly, but not significantly, reduced by DW2008S (Figure 2A). Furthermore, JA, JB and DW2008S reduced Th2 cytokine (IL‐5) levels that had been increased by concanavalin A. The 50% inhibitory concentration (IC_50_) values of JA, JB and DW2008S were 0.16 ± 0.07 μmol/L, 0.24 ± 0.01 μmol/L and 0.73 ± 0.11 μg/mL, respectively (Figure 2B).

To confirm the inhibitory effect of DW2008S on Th2 responses, in vitro Th2 cell polarization was induced in isolated CD4^+^ T cells. The population of CD4^+^GATA3^+^ Th2 cells increased from 3.8% to 38.8% under in vitro Th2 polarization conditions. Furthermore, JA, JB and DW2008S reduced the proportion of CD4^+^GATA3^+^ Th2 cells to 21.4, 15.7 and 18.0%, respectively (Figure 2C). The results imply that the anti‐asthmatic effect of DW2008S and its two active constituents are exerted through Th2‐selective inhibition.

### 3.2 DW2008S negatively regulates TIGIT expression

**Figure 3:**
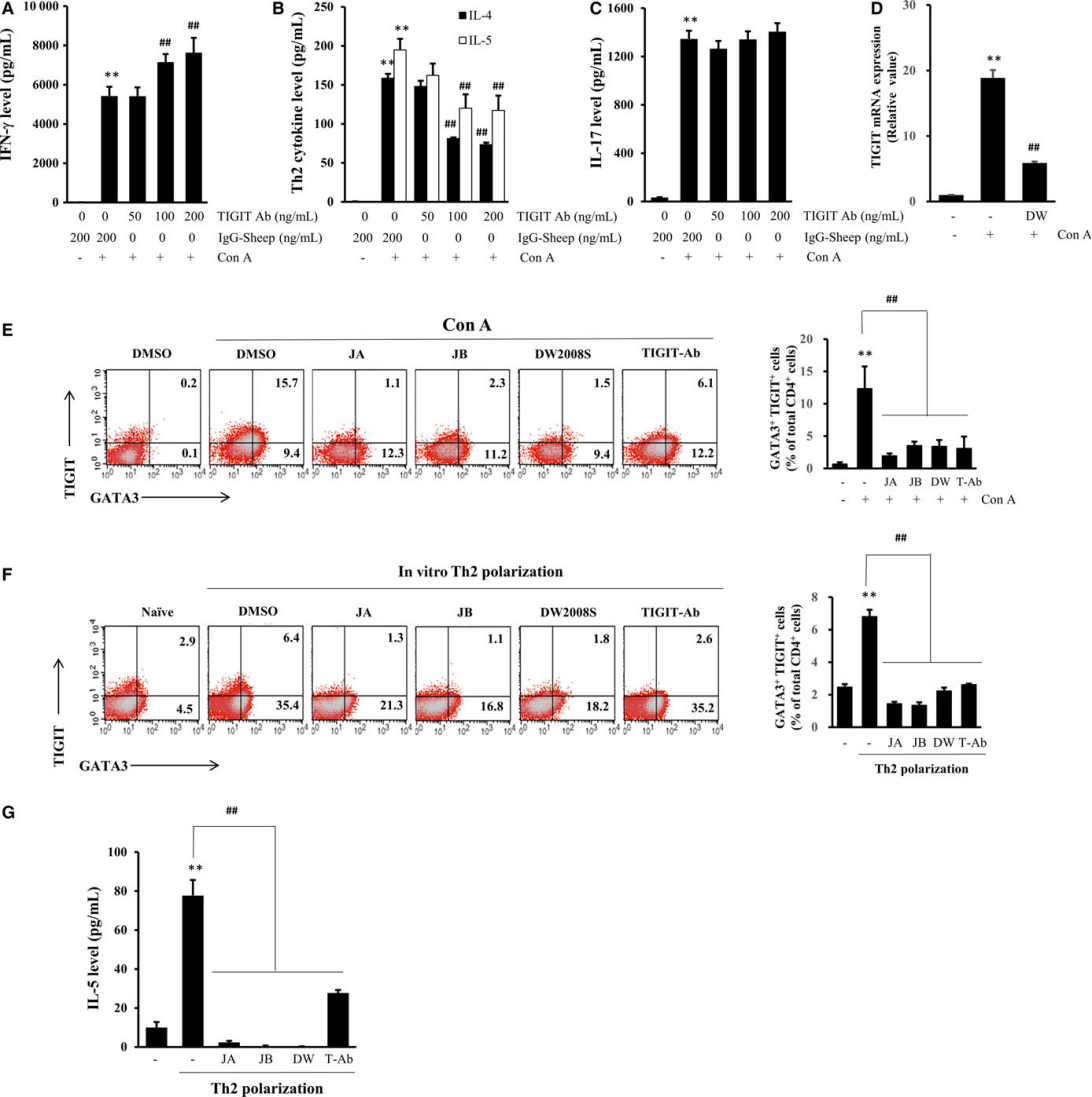
Effects of an anti‐TIGIT neutralizing antibody on Th1/Th2/Th17 cell balance, and effects of DW2008S, JA, and JB on TIGIT expression**.** (A‐C) Spleen cells were cotreated with 5 μg/mL concanavalin A (Con A) and the indicated concentrations of an anti‐TIGIT neutralizing antibody or 200 ng/mL sheep IgG isotype control antibody for 48 h. The levels of (A) Th1 cytokine IFN‐γ, (B) Th2 cytokines IL‐4/5, and (C) Th17 cytokine IL‐17 in the culture media were determined using ELISA kits. (D) Spleen cells were cotreated with 5 μg/mL Con A and 2 μg/mL DW2008S for 48 h. TIGIT mRNA level was determined by RT‐qPCR and normalized to that of β‐actin. (E) Spleen cells were cotreated with 5 μg/mL Con A and 0.5 μmol/L JA, 0.5 μmol/L JB, 2 μg/mL DW2008S, or 100 ng/mL anti‐TIGIT neutralizing antibody for 48 h. Representative fluorescence‐activated cell sorting (FACS) plots for TIGIT ^+^ GATA3^+^ Th2 cells and graphs plotted from the results of at least five independent experiments are shown. (F, G) Isolated CD4^+^ T cells were treated with 0.5 μmol/L JA, 0.5 μmol/L JB, 2 μg/mL DW2008S or 100 ng/mL anti‐TIGIT neutralizing antibody under in vitro Th2‐polarizing conditions. (F) Representative FACS plots for GATA3^+^ Th2 cells and graphs plotted from the results of three independent experiments are shown. (G) IL‐5 level in the culture of in vitro polarized Th2 cells was determined using an IL‐5 ELISA kit. Values are expressed as mean ± SE. ** indicates *P* < .01 when compared to the vehicle, ## indicates *P* < .01 when compared to Con A or the polarized Th2 sample. Ab, antibody; DW, DW2008S; DMSO, dimethyl sulfoxide; T‐Ab, anti‐TIGIT neutralizing antibody

Previous studies have revealed that TIGIT^+^ Treg subsets do not affect Th2 cytokine levels in mouse models of OVA‐induced allergic asthma.^15^ In addition, TIGIT stimulates Th2 cell differentiation through its interaction with CD155 to promote the development of allergic disease.^16^ Therefore, we investigated whether TIGIT is involved in selective regulation of Th2 responses. As shown in Figure 3A‐C, blockade of TIGIT with an anti‐TIGIT neutralizing antibody resulted in selective reduction in Th2 cytokine level in a dose‐dependent manner. However, there was little effect on IL‐17 level, whereas IFN‐γ level increased.

To investigate whether DW2008S is involved in TIGIT regulation, spleen cells were cotreated with concanavalin A and DW2008S, JA, JB, or a neutralizing TIGIT antibody for 48 hours. TIGIT mRNA level was increased by concanavalin A but reduced by DW2008S (Figure 3D). DW2008S and its two major constituents significantly reduced the number of TIGIT^+^ GATA3^+^ Th2 cells in concanavalin A‐activated (Figure 3E) and in vitro polarized Th2 cell populations (Figure 3F). In contrast, DW2008S and its two major constituents did not reduce the number of TIGIT^−^ GATA3^+^ Th2 cells that had been increased by concanavalin A (Figure 3E); however, they significantly reduced the number of cells under in vitro Th2 polarization conditions (Figure 3F). Blockade of TIGIT with an anti‐TIGIT neutralizing antibody did not reduce the number of TIGIT^−^ GATA3^+^ Th2 cells under both conditions. This indicates that DW2008S and its two major constituents may inhibit Th2 differentiation under in vitro Th2 polarization conditions regardless of TIGIT regulation (Figure 3E, F). Interestingly, selective inhibition of TIGIT^+^ GATA3^+^ Th2 cells with the anti‐TIGIT neutralizing antibody without reduction in the number of TIGIT^−^ Th2 cells under in vitro Th2 polarization conditions was sufficient to reduce IL‐5 level, which indicates that TIGIT may be a key factor in mediating Th2 responses (Figure 3G).

We next investigated whether DW2008S could regulate TIGIT expression in Tregs. Our results showed that DW2008S reduced the number of TIGIT^+^ Tregs in the naïve CD4^+^ T cell population (Figure S2A, upper panel; Figure S2B), as well as those under in vitro Treg polarization conditions (Figure S2A, lower panel; Figure S2C). Although DW2008S had a negative effect on the total number of Th2 cells, it had only a little effect on the total number of naïve Tregs. Additionally, it slightly increased the total number of inducible Tregs.

**Figure 4:**
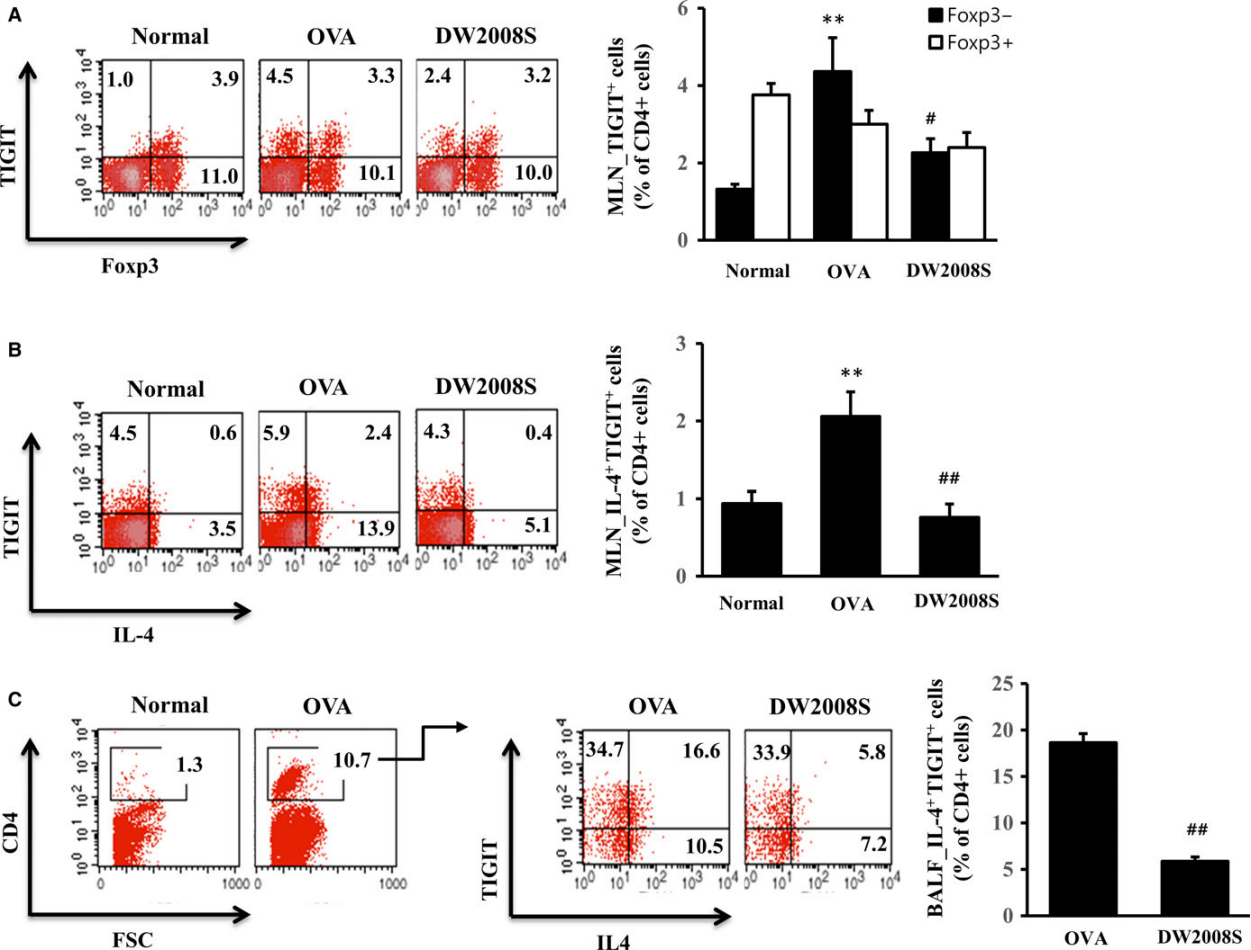
Effects of DW2008 on TIGIT ^+^ Th2 cells in the MLNs and BALF of mice with allergic asthma. BALB/c mice that had been sensitized and challenged with OVA were orally administered 100 mg/kg DW2008S. (A) Fluorescence‐activated cell sorting (FACS) plots and frequencies (%) of MLN TIGIT ^+^ FOXP3^+^ Tregs and MLN TIGIT ^+^ FOXP3^−^ effector cells. (B) FACS plots and frequency (%) of MLN TIGIT ^+^ IL‐4^+^ Th2 cells. (C) FACS plots and frequency (%) of BALF TIGIT ^+^ IL‐4^+^ Th2 cells. Values are expressed as mean ± SE (n* *=* *5). ** indicates *P* < .01 when compared to the Normal group, # indicates *P* < .05 when compared to the OVA group, and ## indicates *P* < .01 when compared to the OVA group

To elucidate whether the selective inhibitory effects of DW2008S on Th2‐driven inflammation arise from TIGIT^+^ Th2 cell regulation or TIGIT^+^ Treg regulation, we evaluated the numbers of subsets of MLN TIGIT^+^ CD4^+^ T cells from mice with allergic airway inflammation.^16^ The total number of MLN TIGIT^+^ CD4^+^ T cells was significantly higher in the OVA asthma group than in the Normal group; however, it was normalized after DW2008S was administered to the animals (Figure 4A, B). Interestingly, MLN TIGIT^+^ FOXP3^+^ Tregs represented > 80% of the total MLN TIGIT^+^ CD4^+^ T cells in the normal mice. Only TIGIT^+^FOXP3^−^ effector cells increased in number in the OVA asthmatic mice. However, this effect was attenuated by DW2008S, suggesting that the anti‐asthmatic effects of DW2008S are related to TIGIT^+^FOXP3^−^ effector cells and not TIGIT^+^FOXP3^+^ Tregs (Figure 4A). The number of MLN TIGIT^+^IL‐4^+^ Th2 cells increased in the OVA asthmatic mice but was reduced by DW2008S (Figure 4B).

In addition, we investigated BALF TIGIT^+^ IL‐4^+^ Th2 cells. The results showed that CD4^+^ lymphocytes were undetectable in the BALF samples of normal mice; however, they increased in number in the BALF samples of OVA asthmatic mice (Figure 4C). In the latter group, TIGIT^+^ cells formed ~50% of the total cell number, indicating that their proportion was much higher than that of MLN cells. Furthermore, TIGIT^+^ IL‐4^+^ Th2 cells formed 18.6 ± 1.0% of the total cell number. DW2008S significantly reduced the proportion of BALF TIGIT^+^ IL‐4^+^ Th2 cells to 5.9 ± 0.5%. Our results indicate that the selective inhibitory effects of DW2008S on Th2 responses may result from the regulation of TIGIT^+^ Th2 cell number in mice with asthma.

### 3.3 DW2008S selectively inhibits A_3_ AR and PDE4

**Figure 5:**
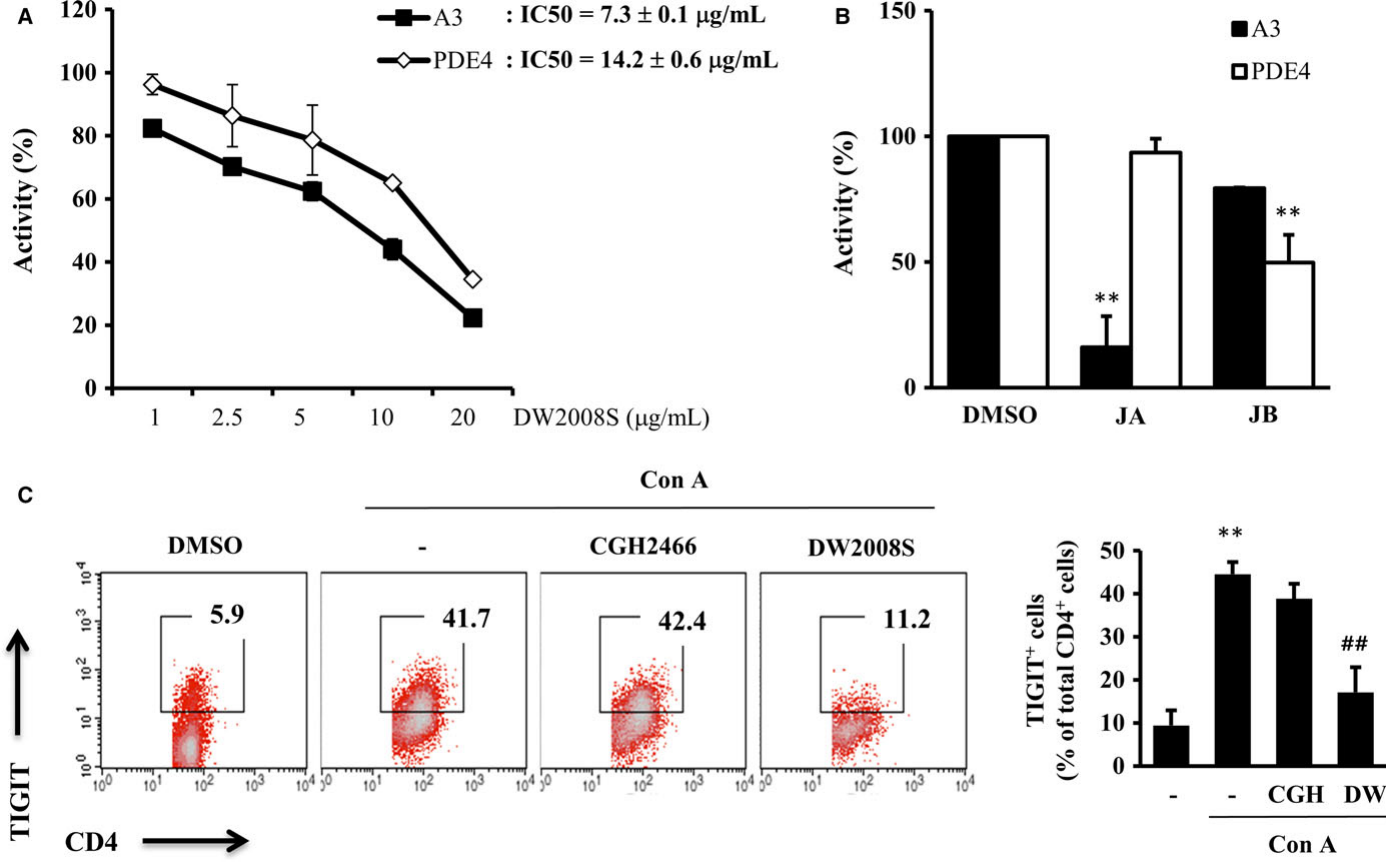
Effects of DW2008S, JA and JB on A_3_ AR and PDE4 activities. A_3_ AR activity was determined by a radioligand‐binding assay using [^125^I]‐AB‐MECA. PDE4 activity was determined by an enzyme assay using a [^3^H]‐adenosine substrate. (A) Effects of DW2008S on A_3_ AR and PDE4 activities. (B) Effects of JA (2 μmol/L) and JB (2 μmol/L) on A_3_ AR and PDE4 activities. (C) Spleen cells were cotreated with 5 μg/mL concanavalin A (Con A) and 1 μmol/L CGH2466 (CGH) or 2 μg/mL DW2008S (DW) for 48 h. Representative fluorescence‐activated cell sorting plots for TIGIT ^+^ CD4^+^ cells and graphs plotted from the results of three independent experiments are shown. ** indicates *P* < .01 when compared to the vehicle and ## indicates *P* < .01 when compared to Con A. DMSO, dimethyl sulfoxide

To determine whether DW2008S and its constituents affect other targets in addition to TIGIT, we performed screening assays of 85 targets related to allergic, respiratory and inflammatory diseases. The results showed that DW2008S inhibited A_3_ AR and PDE4 activities (Table 1). Additionally, 20 μg/mL DW2008S antagonized A_3_ AR activity by 96% but did not antagonize other AR isoforms in the FLIPR calcium flux assay (Table 1). The treatment also antagonized A_3_ AR activity (IC_50_ value, 7.25 ± 0.05 μg/mL) in the radioligand‐binding assay with [^125^I]‐AB‐MECA. Moreover, DW2008S inhibited PDE4 activity (IC_50_ value, 14.2 ± 0.6 μg/mL) in the enzyme assay with [^3^H]‐adenosine substrate (Figure 5A). JA and JB were also tested in these assays to determine whether they are associated with A_3_ AR antagonism and PDE4 inhibition. Interestingly, JA (2 μmol/L) selectively antagonized A_3_ AR activity, whereas JB (2 μmol/L) selectively inhibited PDE4 activity. The indicated concentrations of JA and JB were higher than the respective concentrations required to inhibit Th2 responses (Figure 5B). Treatment of activated spleen cells with CGH2466, a dual inhibitor of A_3_ AR and PDE4, induced little changes in TIGIT^+^ CD4^+^ cell numbers, indicating that the inhibitory activities of DW2008S on A_3_ AR and PDE4 may not affect TIGIT regulation (Figure 5C).

**Table 1:** Target screening results

Target family	Target	Activation (%)	Inhibition (%)	Target family	Target	Activation (%)	Inhibition (%)
Acetylcholine receptor	M_1_	−1.4	−7.3	Lipoxygenase	5‐LO	–	4
	M_2_	0.2	−1.3		12‐LO	–	12
	M_3_	−4.4	13.8		15‐LO	–	39
Adenosine receptor	A_1_	−4.0	−15.4	Lysophospholipid receptor	S1P1	3.1	14.8
	A_2A_	0.2	−5.4		S1P2	−3.0	−3.3
	A_2B_	2.8	29.3		S1P3	1.1	9.6
	A_3_	7.1	96		S1P4	4.4	−19.2
Adrenoceptor	ADRB2	−6.1	4.7		S1P5	−1.6	−2.0
Anaphylatoxin receptor	C3AR	10.2	−13.9	Neuromedin U receptor	NMU1	−0.4	−1.3
	C5AR	−5.1	−3.9	Neuropeptide Y receptor	Y2	−1.3	–
Bombesin receptor	BB_2_	−4.9	20.7	*N*‐formylpeptide receptor	FPR1	−0.2	10.3
Bradykinin receptor	BDKR2	−5.3	−0.7	Nicotinic acid receptor	GPR109A	−2.5	5.9
Calcitonin receptor	CGRP1	−0.7	7.4	Opioid receptor	OPRD1	0.6	15.1
Cannabinoid receptor	CB_1_	–	−9.7		OPRK1	−9.4	13.3
	CB_2_	−0.2	34.7		OPRM1	−4.8	−1.6
Cholecystokinin receptor	CCK_2_	–	−7.3	Phosphodiesterase	PDE2	–	4
Chemoattractant receptor	CMKLR1	−5.1	−6.9		PDE3	–	19
Chemokine receptor	CCR1	−0.3	5.9		PDE4	–	65
	CCR10	2.4	−6.0	Platelet‐activating factor receptor	PAF	2.6	−26.8
	CCR2B	−0.4	16.3	Prolactin‐releasing peptide receptor	PRP	−5.2	–
	CCR3	−2.5	−14.3	Prokineticin receptor	PK1	2.2	−172.1
	CCR4	−1.9	−12.8	Tachykinin/neurokinin receptor	NK1	−2.4	−15.0
	CCR5	−1.6	−7.5		NK2	0.1	2.3
	CCR6	−0.4	3.2		NK3	−2.4	−172.8
	CCR7	0.0	−11.5	Prostanoid receptor	DP	6.4	−33.3
	CCR8	1.0	0.6		EP1	−2.6	6.7
	CCR9	−1.2	−5.5		EP2	−1.1	24.2
	CX3CR1	4.2	−7.3		EP3	−6.9	25.0
	CXCR1	−3.7	−12.3		IP1	−1.2	2.5
	CXCR2	22.4	16.0		CRTH2	–	6
	CXCR3	1.3	−14.3		TP	−3.4	−18.6
	CXCR4	−1.6	−14.3	Protease‐activated receptor	Trypsin‐activated PARs	−3.5	4.6
	CXCR6	5.8	−9.2		Thrombin‐activated PARs	−0.6	−1.7
	XCR1	0.7	21.5	Protein tyrosine kinase	LYNA	–	−13
Free fatty acid receptor	GPR43	−1.3	−5.9		LYNB	–	−7
GABA_B_ receptor	GABAB1b	6.5	−45.1	Parathyroid hormone receptors	PTH1	0.7	–
Galanin	GAL1	−4.2	–	Serotonin receptor	5‐HT_2A_	2.7	−1.8
Gonadotropin‐releasing hormone receptor	GnRH	−1.7	–	Somatostatin receptor	SST4	−2.3	–
Histamine receptor	H_1_	0.3	−0.4	VIP/PACAP receptor	PAC1	−0.3	11.9
	H_2_	−1.3	−1.1		VPAC1	−5.7	−0.1
Leukotriene receptor	BLT1	−6.2	−0.9		VPAC2	1.4	−5.5
	CYSLTR1	−2.1	5.3	Xanthine oxidase	XO	–	9
	CYSLTR2	−10.9	−45.6				

–, Not tested.

Concentration of DW2008S: agonist test, 25 μg/mL; antagonist test, 20 μg/mL.

GABA, gamma‐aminobutyric acid; PACAP, pituitary adenylate cyclase‐activating polypeptide; VIP, vasoactive intestinal peptide.

**Figure 6:**
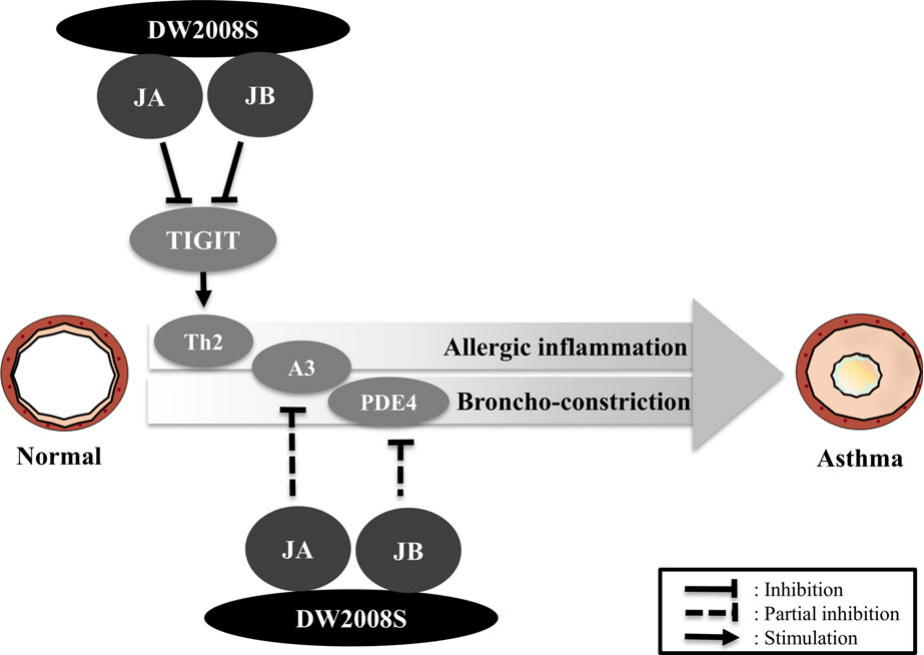
Schematic presentation of the mechanism underlying the protective effect of DW2008S against allergic asthma

These results suggest that DW2008S plays a critical role in protecting against airway allergic inflammation and bronchoconstriction through negative regulation of TIGIT and blockade of A_3_ AR and PDE4 activities (Figure 6).

## 4 DISCUSSION

Recent studies have provided evidence that TIGIT is an important factor in Th2‐driven responses and Th1/Th2/Th17 cell balance.^16^, ^25^ However, no regulators of TIGIT, except for a few specific antibodies, have been identified or developed. We initially found that DW2008S selectively suppressed Th2 cytokines but not Th1/Th17 cytokines in concanavalin A‐activated spleen cells. These results indicate that DW2008S possibly regulates TIGIT expression. Furthermore, DW2008S and its two major constituents significantly reduced TIGIT expression in both concanavalin A‐activated spleen cells and polarized Th2 cells isolated from CD4^+^ T cells. Consistent with the results of a previous study, which showed increased TIGIT expression in the MLNs of mice with Th2‐driven allergic asthma,^16^ we observed that TIGIT expression was elevated in the MLNs of mice with allergic asthma. However, TIGIT expression was restored to its normal level by DW2008S. The increase in TIGIT expression and its attenuation by DW2008S in mice with allergic asthma appear to be limited to FOXP3^−^ effector T cells, which seems to mostly result from fluctuations in number of TIGIT^+^ IL‐4^+^ Th2 cells.

TIGIT^+^ Th2 cells may substantially contribute to the secretion of Th2 cytokines. DW2008S, JA, JB and an anti‐TIGIT neutralizing antibody reduced Th2 cytokine expression and the number of TIGIT^+^ GATA3^+^ Th2 cells but not TIGIT^−^ GATA3^+^ Th2 cells in the concanavalin A‐activated spleen cells. In the in vitro Th2 cell polarization test, the anti‐TIGIT neutralizing antibody also reduced the level of IL‐5 without changing the number of TIGIT^−^ GATA3^+^ Th2 cells. The results are in agreement with those of previous studies, which shows that sorted TIGIT^+^ Th2 cells secrete higher amounts of Th2 cytokines than TIGIT^−^ Th2 cells do.^16^

Tregs are well known to protect against the progression of asthma.^26^, ^27^ However, Tregs are also associated with autoimmune diseases such as rheumatoid arthritis, which is reminiscent of Th1/Th17‐driven inflammation.^28^ Moreover, TIGIT^+^ Tregs selectively suppress Th1/Th17 cell responses while sparing or even promoting Th2 cell responses. In contrast, TIGIT^−^ Tregs can suppress all Th1/Th2/Th17 cell responses, although they exert lower suppressive effects than TIGIT^+^ Tregs do.^15^ Regarding in vitro Treg polarization, DW2008S selectively reduced the number of TIGIT^+^ Tregs without changing the total number of Tregs. However, the number of MLN FOXP3^+^ Tregs, including TIGIT^+^ Tregs, in the mice with allergic asthma did not increase compared to the number in the normal mice. Additionally, their numbers were only slightly changed by DW2008S. Regarding Tregs, the discrepancy between the in vitro and in vivo observations may be due to tissue and/or stimulator differences. The results indicate that DW2008S may ameliorate allergic asthma through regulation of TIGIT expression in Th2 cells rather than in Tregs.

In this study, we showed that the concentrations of DW2008S required to inhibit the activities of A_3_ AR and PDE4 are higher than that required to regulate TIGIT expression. Although ARs and PDE isoforms are associated with respiratory diseases such as asthma and COPD, their non‐selective blockade can cause adverse effects related to cardiac and central nervous system functions. Therefore, selective antagonism of A_3_ AR by DW2008S and JA and selective inhibition of PDE4 by DW2008S and JB may inhibit airway inflammation and enhance bronchodilation without causing adverse effects.

Regulation of multiple targets has been shown to result in additive or synergistic effects and may be effective in preventing and treating heterogeneous diseases.^29^ It is apparent from our results that DW2008S can prevent allergic airway inflammation and bronchoconstriction via three different mechanisms. Based on the inhibitory concentrations of DW2008S and those of its major constituents for the three targets, negative regulation of TIGIT expression is likely to be the main contributor to the anti‐asthmatic properties of DW2008S, whereas blockade of A_3_ AR and PDE4 activities may be a partial contributor at higher DW2008S concentrations. Our results demonstrate that DW2008S effectively ameliorates allergic airway inflammation and bronchoconstriction via negative regulation of TIGIT expression and blockade of A_3_ AR and PDE4 activities.

## CONFLICTS OF INTERESTS

The authors declare that they have no conflicts of interests.

## AUTHOR CONTRIBUTIONS

All the authors designed the experiments and contributed to the preparation of the manuscript. H. Lee, J. Youm and J. Yoon performed the experiments. Y.W. Choi and J. Yoon analysed the data. J. Yoon wrote the manuscript.
