# Supplementary material for: DW2008S and its major constituents from Justicia procumbens exert anti‐asthmatic effect via multitargeting activity
Source: J Cell Mol Med. 2018 Mar 7;22(5):2680–91. doi: 10.1111/jcmm.13550 (PMC5908124; doi:10.1111/jcmm.13550)

**Supporting information**

**Fig. S1. HPLC profile of DW2008S.**

**Fig. S2. Effects of DW2008S on TIGIT^+^ Tregs in isolated naïve CD4^+^ T cells and *in vitro* polarized Tregs.** Isolated CD4^+^ T cells were treated with 2 μg/mL DW2008S, 100 ng/mL anti-TIGIT neutralizing antibody, or 100 ng/mL anti-sheep IgG antibody (negative control) in the presence or absence of 5 ng/mL transforming growth factor (TGF)-β1 for 4 days. (A) Representative fluorescence-activated cell sorting plots for TIGIT^+^ Foxp3^+^ Tregs. (B) Graphs showing the percentages of TIGIT^+^ and TIGIT^−^ Tregs in the population of naïve CD4^+^ T cells. (C) Graphs showing the percentages of TIGIT^+^ and TIGIT^−^ cells among *in vitro* polarized Tregs. Data are expressed as mean ± SE. ** indicates p < 0.01 when compared to the vehicle, whereas ## indicates p < 0.01 when compared to the polarized Treg sample.

Fig. S1.


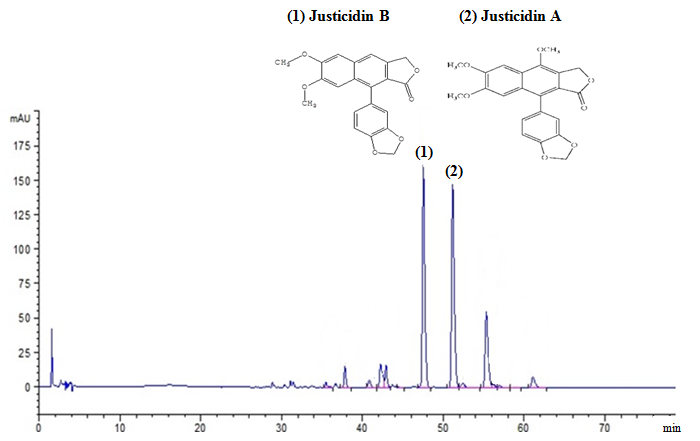


Fig S2.


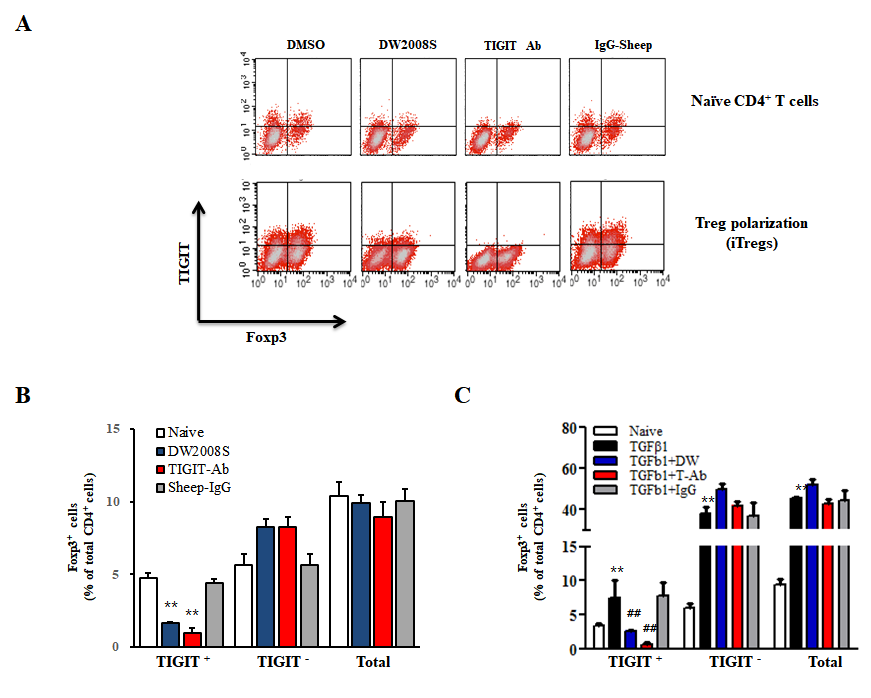

Supplement: Supplementary file 1 [file JCMM-22-2680-s001.docx]
